# Supplementary material for: Benefit-cost analysis of the policy of mandatory annual rabies vaccination of domestic dogs in rabies-free Japan
Source: PLoS One. 2018 Dec 17;13(12):e0206717. doi: 10.1371/journal.pone.0206717 (PMC6296744; doi:10.1371/journal.pone.0206717)
Supplement: S1 Table — (DOCX) [file pone.0206717.s001.docx]

***PLOS ONE***

**Benefit-cost analysis of the policy of mandatory annual rabies vaccination of domestic dogs in rabies-free Japan**

Nigel C. L. Kwan, Akio Yamada, Katsuaki Suigiura

Supporting information

S1 Table. List of cost data and input variables included in the current benefit-cost analysis.

| **Parameter** | **Probability distribution / fixed value** | **Unit** | **Estimated mean (90% prediction interval) / estimated fixed value** | **Source / Explanation** |
| --- | --- | --- | --- | --- |
| **Annual costs of implementing current rabies vaccination policy in Japan (*Costs*_annual_)** | | | | |
| ***Direct costs* = *N*_vac_ X *Cost*_vac_** | | | | |
| Number of registered companion dogs vaccinated with rabies in 2015 (*N*_vac_) | 4,688,240 | dog | Not applicable (n/a) | 2015 Ministry of Health, Labour and Welfare data (MHLW, 2017) |
| Single rabies vaccination cost (*Cost*_vac_) | $29.52 | US dollar | n/a | Proxy inferred from the weighted mean of the prices charged at vaccination campaign in the 47 prefectures of Japan |
| ***Indirect costs* = *N*_owner_ X *T*_lost,vac_ X *GDP*_lost_ + *N*_drive_ X *D*_clinic_ X *Cost*_fuel_ + *Cost*_ad_** | | | | |
| *Time (income) loss* | | | | |
| Annual number of owners vaccinating their dogs (*N*_owner_) | *N*_vac_ ÷ 1.24 | person | 3,780,839 | Calculated from 2015 national survey information that a single household owns on average 1.24 companion dogs (JPFA, 2018) |
| Number of working days lost per owner per dog vaccination (*T*_lost,vac_) | *Uniform* (0.5 , 2) ÷ 24 | day | 0.05  (0.02 – 0.08) | Authors’ assumption |
| Daily gross domestic product per capita (*GDP*_lost_) | $105.31 | US dollar | n/a | 2017 IMF data |
| *Transport costs* | | | | |
| Number of owners vaccinating their dogs at clinic and travelling by car (*N*_drive_) | *N*_owner_ X *Uniform* (0.514 , 0.528) X 0.5 | person | 984,908  (972,998 – 996,817) | Assuming 51.4% to 52.8% of owners would vaccinate their dogs at clinic and 50% of them would drive; no transport costs were considered for owners attending vaccination campaign and for owners who walk to clinic |
| Driving distance of a return trip to an animal clinic (*D*_clinic_) | $2 X \sqrt{377,962 \div11,839 \div\pi}$ | km | 6.38 | Estimation based on the area of Japan, i.e. 377,962 km^2^, and the reported number of companion animal clinics in Japan, i.e. 11,839 (MAFF, 2018) |
| Fuel cost per km (*Cost*_fuel_) | $0.21 | US dollar | n/a | Authors’ assumption |
| *Advertisement costs* (*Cost*_ad_) | $4,333 | US dollar | n/a | 2015 MHLW data |
| **Economic burden of a dog rabies outbreak in Japan under current annual vaccination policy (*Burden*_vac_) and under the abolition of such policy (*Burden*_abolish_)*** | | | | |
| ***Dog rabies control costs* = *Emergency dog vaccination costs* + *Stray dog depopulation and epidemiological investigation costs*** | | | | |
| Mean number of rabies cases (*N*_case_) | 4.7 | dog | n/a | Simulated outbreak in Ibaraki Prefecture (Kadowaki et al, 2018) |
|  | 21.7 |  |  |  |
| Mean epidemic period (*D*_rabies_) | 68.2 | day | n/a | Simulated outbreak in (Kadowaki et al, 2018) |
|  | 152.5 |  |  |  |
| Days of action (*D*_action_) | *D*_rabies_ – 30 + 40 | day | 78.2 | Assuming a 30-day delay in action and that rabies control actions would continue for another 40 days after the last case was observed based on the field experience of Malaysia outbreak (Bamaiyi, 2015; Kadowaki et al, 2018) |
|  |  |  | 162.5 |  |
| ***Emergency dog vaccination costs* = *N*_e-vac_ X *Cost_vac_*** | | | | |
| Daily number of dogs receiving emergency vaccination (*N*_e-vac_) | *Poisson* (100) | dog | 100 (84 – 117) | Assumption based on Kadowaki et al (2018) |
| ***Stray dog depopulation and epidemiological investigation costs* = *D*_action_ X (*N*_officer_ X *Cost*_labour_ + *N*_car_ X *Cost*_drive_ + *N*_stray_ X *Cost*_stray_)** | | | | |
| Additional government officers in the rabies control team (*N*_officer_) | 50 | person | n/a | Assumption based on the information from the investigated prefectural governments |
|  | 100 |  |  |  |
| Daily labour cost of an officer of the rabies control team (*Cost*_labour_) | $108.01 | US dollar | n/a | Proxy based on the average monthly salary of prefectural public servants (2017 Ministry of Internal Affairs and Communications data) |
| Daily increase in the number of car vehicles used for capturing stray dogs and epidemiological investigation (*N*_car_) | 15 | car | n/a | Assumption based on the information from the investigated prefectural governments |
|  | 30 |  |  |  |
| Driving cost per car (*Cost*_drive_) | 50 X 0.21 | US dollar | $10.7 | Assuming a mean travel distance of 50 km and $0.21 for fuel cost per km |
| Daily increase in the number of stray dogs captured (*N*_stray_) | *Poisson* (5) | dog | 5 (2 – 9) | Assumption based on the information from the investigated prefectural governments |
| Costs of care and euthanasia for each captured stray dog (*Cost*_stray_) | *Uniform* (118 , 155) | US dollar | $136  ($120 – 153) | Assumption based on the information from the investigated prefectural governments |
| ***Surveillance costs* = *N*_survey_ X *Cost*_test_** | | | | |
| Duration of active surveillance during and after the outbreak (*D*_survey_) | *D*_action_ + 730 | day | 808 | Testing of suspected rabid animals for another two years to declare rabies-free status according to OIE standards |
|  |  |  | 893 |  |
| Daily number of suspected rabid animals tested under active surveillance (*N*_d-survey_) | *Poisson* (3) | animal | 3 (1 – 6) | Assumption based on the surveillance data of France (2017 Rabies Bulletin Europe data) |
|  | *Poisson* (5) |  | 5 (2 – 9) |  |
| Total number of animals tested including rabid dogs and suspected animals (*N*_survey_) | *N_case_* + *D*_survey_ X *N*_d-survey_ | animal | 2429  (813 – 4854) | n/a |
|  |  |  | 4,484  (1,807 – 8,054) |  |
| Costs of single diagnostic testing including direct fluorescent antibody test and RT-PCR (*Cost*_test_) | $178 | US dollar | n/a | Assumption based on the information from the investigated prefectural governments |
| ***Human rabies prevention costs, i.e. post-exposure prophylaxis (PEP) and pre-exposure prophylaxis (PrEP) costs*** | | | | |
| ***Direct costs*** | | | | |
| ***PEP costs due to rabid dog exposure* = *N*_PEP_ X (*Cost*_h-vac_ X 5 + *P*_III_ X *Cost*_RIG_)** | | | | |
| Number of patients receiving PEP due to rabid dog-bite injury (*N*_PEP_) | 6.7 | person | 6.7 | Assumption based on [4] and estimation based on the ratio of *N*_case,abolish_ : *N*_case,vac_ which is 4.62 : 1 |
|  | 6.7 X 4.62 |  | 30.9 |  |
| Cost of single rabies vaccination (*Cost*_h-vac_) | $129 | US dollar | n/a | Proxy based on the price of Japanese PCEC-K vaccine charged at human hospital (2018 Tokyo Metropolitan Cancer and Infectious Disease Center Komagome Hospital data) |
| Probability of patients having a Category III exposure and requiring rabies immunoglobulin (RIG), i.e. proportion of Category III exposure among Category II and III exposures caused by rabid dogs (*P*_III_) | *Uniform* (0.5 , 0.72) | n/a | 0.61  (0.51 – 0.71) | Van Rijckevorsel et al., 2012; Tsiodras et al. 2013 |
| Cost of single RIG (*Cost*_RIG_) | Uniform (600 , 1200) | US dollar | $900  ($630 – 1,170) | De Benedictis et al. (2016) |
| ***PEP costs due to public panic* = *N*_panic_ X *D*_panic_ X *Cost*_h-vac_ X 5** | | | | |
| Daily number of people receiving PEP for animal bites or scratches due to panic (*N*_panic_) | *Poisson* (6) | person | 6  (2 – 10) | Assumption based on the reported average daily incidence of dog-bite victims in Japan which is 12 persons (Ministry of the Environment, 2017) |
|  | *Poisson* (10) |  | 10  (5 – 15) |  |
| Duration of panic behavior (*D*_panic_) | *D*_action_ | day | 78 | Assumption based on the field experience of the Taiwan outbreak (Huang et al., 2013) |
|  |  |  | 163 |  |
| ***Occupational PrEP costs* = *N*_officer_ X *Cost*_h-vac_ X 3** | | | | |
| ***Indirect costs*** | | | | |
| ***Time (income) loss* (for PEP due to rabid dog exposure) = *N*_PEP_ X (1 + *P*_child_) X 5 X *T*_lost_ X *GDP*_lost_**  ***Time (income) loss* (for PEP due to public panic) = *N*_panic_ X (1 + *P*_child_) X 5 X *T*_lost_ X *GDP*_lost_** | | | | |
| Proportion of child patients receiving PEP accompanied by another adult (*P*_child_) | *Beta* (83 + 1 , 235 – 83 + 1) | person | 0.35  (0.3 – 0.41) | Sudarshan et al., 2007 |
| Time lost per visit (*T*_lost_) | 0.5 | day | n/a | Knobel et al., 2005 |
| ***Transport costs* (assuming half of the people receiving PEP would drive and the other half would take public transport)**  ***Driving costs* (for PEP due to rabid dog exposure) = 0.5 X *N*_PEP_ X 5 X *D*_drive_ X *Cost*_fuel_**  ***Driving costs* (for PEP due to public panic) = 0.5 X *N*_panic_ X 5 X *D*_drive_ X *Cost*_fuel_**  ***Public transport costs* (for PEP due to rabid dog exposure) = 0.5 X *N*_PEP_ X (1 + *P*_child_) X 5 X *Cost*_transport_**  ***Public transport costs* (for PEP due to public panic) = 0.5 X *N*_panic_ X (1 + *P*_child_) X 5 X *Cost*_transport_** | | | | |
| Driving distance per return trip (*D*_drive_) | $2 X \sqrt{377,962 \div114 \div\pi}$ | km | 65 | Estimation based on the area of Japan, i.e. 377,962 km^2^, and the reported number of hospitals and clinics providing PEP and PrEP, i.e. 114 (2018 MHLW [FORTH] data) |
| Public transport fare per return trip (*Cost*_transport_) | $8.92 | US dollar | n/a | Authors’ assumption |
| **Probability input into the chance nodes of the decision tree model** | | | | |
| Annual probability of rabies introduction into Japan (*P_annual*) | 2.57 X 10^-5^ | n/a | n/a | Kwan et al., 2017 |

*When a table cell is split by a dashed line, the upper row presents information on a dog rabies outbreak under the current vaccination policy, while the bottom row presents information on an outbreak under the abolition of vaccination policy

References

Bamaiyi, P.H. 2015. Outbreak of Canine Rabies in Malaysia: Review, Analysis and Perspectives. J. Vet. Adv. 5, 1181–1190.

De Benedictis, P., Minola, A., Nodari, E.R., Aiello, R., Zecchin, B., Salomoni, A., Foglierini, M., Agatic, G., Vanzetta, F., Lavenir, R. and Lepelletier, A. 2016. Development of broad‐spectrum human monoclonal antibodies for rabies post‐exposure prophylaxis. EMBO molecular medicine 8, 407–421.

Huang, J.J., You, K.H., Lin, C.F., Wang, C.Y., Liu, C.L., Yen, J.J. 2013. 2013 Taiwan's Strategies in Response to Re-emergence of Animal Rabies. Taiwan Epidemiology Bulletin 29.

Kadowaki, H., Hampson, K., Tojinbara, K., Yamada, A., Makita, K. 2018. The risk of rabies spread in Japan: a mathematical modelling assessment. Epidemiol. Infect. 146, 1245–1252.

Knobel, D.L., Cleaveland, S., Coleman, P.G., Fèvre, E.M., Meltzer, M.I., Miranda, M.E.G. Shaw, A., Zinsstag, J., Meslin, F.X. 2005. Re-evaluating the burden of rabies in Africa and Asia. Bulletin of the World Health Organization 83, 360–368.

JPFA – Japan Pet Food Association 2018. National surveys of pet dogs and cats [in Japanese], http://www.petfood.or.jp/data/index.html (accessed 2 July 2018).

Kwan, N.C.L., Sugiura, K., Hosoi, Y., Yamada, A., Snary, E.L. 2017a. Quantitative risk assessment of the introduction of rabies into Japan through the importation of dogs and cats worldwide. Epidemiol. Infect. 145, 1168–1192.

MAFF – Ministry of Agriculture, Forestry and Fisheries 2018. Situation of animal care facilities in 2017 [in Japanese], http://www.maff.go.jp/j/tokei/kouhyou/animal/attach/pdf/index-2.pdf (accessed 2 July 2018).

MHLW – Ministry of Health, Labour and Welfare 2017. Number of dogs registered and vaccinated against rabies (1960–2016) [in Japanese], http://www.mhlw.go.jp/bunya/kenkou/kekkaku-kansenshou10/02.html (accessed 2 July 2018).

Ministry of the Environment 2017. National dog-bite incidence (1974–2016), https://www.env.go.jp/nature/dobutsu/aigo/2_data/statistics/gyosei-jimu_h29.html (accessed 2 July 2018).

Sudarshan, M.K., Madhusudana, S.N., Mahendra, B.J., Rao, N.S.N., Narayana, D.A., Rahman, S.A., Meslin, F.X., Lobo, D., Ravikumar, K. 2007. Assessing the burden of human rabies in India: results of a national multi-center epidemiological survey. Int. J. Infect. Dis. 11, 29–35.

Tsiodras, S., Dougas, G., Baka, A., Billinis, C., Doudounakis, S., Balaska, A. 2013. Re-emergence of animal rabies in northern Greece and subsequent human exposure, October 2012–March 2013. Euro. Surveill. 18.

Van Rijckevorsel, G.G., Swaan, C.M., Van Den Bergh, J.P., Goorhuis, A., Baayen, D., Isken, L., Timen, A., van den Hoek, A. 2012. Rabid puppy-dog imported into the Netherlands from Morocco via Spain, February 2012. Euro. Surveill. 17.
